# Frequency and survival of delayed breast cancer diagnosis in women participating at screening mammography in the Netherlands: a population-based study

**DOI:** 10.1016/j.lanepe.2025.101526

**Published:** 2025-11-14

**Authors:** Lucien E.M. Duijm, Eline L. van der Veer, Hermen C. van Beek, Wikke Setz-Pels, Vivian van Breest Smallenburg, Rob M.G. van Bommel, Clemence L. op de Coul-Froger, Maaike Gielens, Dominique J.P. van Uden, Adri C. Voogd

**Affiliations:** a Department of Radiology, Canisius Wilhelmina Hospital, Weg Door Jonkerbos 100, 6532 SZ, Nijmegen, the Netherlands; b Department of Radiology, Erasmus Medical Centre, Dr. Molewaterplein 40, 3015 GD, Rotterdam, the Netherlands; c Department of Radiology, Maxima Medical Centre, De Run 4600, 5504 DB, Veldhoven, the Netherlands; d Department of Radiology, Catharina Hospital, Michelangelolaan 2, 5623 EJ, Eindhoven, the Netherlands; e Department of Radiology, Jeroen Bosch Hospital, Henri Dunantstraat 1, 5223 GZ, ‘s-Hertogenbosch, the Netherlands; f Department of Radiology, St Anna Hospital, Bogardeind 2, 5664 EH, Geldrop, the Netherlands; g Department of Radiology, Bernhoven Hospital, Nistelrodeseweg 10, 5406 PT, Uden, the Netherlands; h Department of Surgical Oncology, Canisius Wilhelmina Hospital, Weg Door Jonkerbos 100, 6532 SZ, Nijmegen, the Netherlands; i Department of Epidemiology, Maastricht University, Peter Debeyeplein 1, 6229 HA, Maastricht, the Netherlands

**Keywords:** Breast cancer, Diagnostic delay, Screening programme, Tumour characteristics

## Abstract

**Background:**

Although breast cancer screening programmes aim to enable early breast cancer detection, diagnostic delays still occur among participants. Limited information is available on the frequency and survival of women with a delay in breast cancer diagnosis within the screening population. We determined the frequency of various types of delay in breast cancer diagnosis at screening mammography and specified the tumour characteristics, surgical therapy and survival rates of women with these delayed diagnoses, as well as variations in their proportions over time.

**Methods:**

We included 901,133 screening examinations obtained in the southern Netherlands between 1999 and 2019. Screening mammograms of women with interval cancers (ICs) and breast cancers detected at subsequent screening were reviewed to determine whether the cancer had been missed.

**Findings:**

Of the 7129 women with breast cancers, 5419 (76.0%) were diagnosed without delay after recall and 1101 (15.4%) had a true IC (i.e., not detectable at the previous screen). In total, 1601 women experienced a delay in breast cancer diagnosis, comprising the following three study groups: (i) recalled women with a delay in diagnostic work-up (n = 264), (ii) recalled women with screen-detected cancers (SDCs) at subsequent screening and without a delay in diagnostic work-up, that had been missed at the previous screening round (n = 992), and (iii) women with ICs missed at the latest screening round (n = 345). Overall, 26.6% of cancers were associated with a delay (1601/6028), primarily due to SDCs missed at the previous screen (62.0%, 992/1601), followed by missed ICs (21.5%, 345/1601) and misdiagnosis after recall (16.5%, 264/1601). Compared to SDCs missed at the previous screen and misdiagnosis after recall, missed ICs demonstrated the poorest tumour characteristics, highest mastectomy rate (42.6% vs 20.1% and 19.3%, p < 0.0001) and poorest overall survival (5-year rate 86.9% vs 93.8% and 93.8%, p = 0.0017). Temporal trends in tumour characteristics were mainly observed in SDCs missed at the previous screen.

**Interpretation:**

Delayed breast cancer diagnosis at screening mammography or after recall remains a serious point of concern. Most delays are related to SDCs missed at a previous screen, whereas missed ICs show the worst survival.

**Funding:**

This research did not receive any funding.

**Research in context**

**Evidence before this study**

Screening is intended to detect breast cancer at an early stage; however, some women undergoing screening mammography may experience significant delays in diagnosis. To identify evidence on this issue, we searched PubMed using the MeSH terms “breast neoplasm” and/or “cancer screening,” restricting results to articles published from 2000 onwards. The literature mainly addresses the causes of diagnostic delay among screened women, including cancers overlooked at screening, incorrect BI-RADS classification during diagnostic work-up, and misinterpretation of breast imaging findings by radiologists. Only one Norwegian study has examined survival outcomes, reporting no significant difference between delayed and timely diagnoses. To date, no study has evaluated the proportions of different types of diagnostic delay or their impact on survival within the screened population.

**Added value of this study**

We investigated the degree to which women participating in the Dutch population-based screening programme experienced delays in breast cancer diagnosis after recall, or were diagnosed with screen-detected or interval breast cancers missed at their prior biennial screening or most recent screen. Additionally, we assessed the tumour characteristics, surgical treatments, and overall survival rates of these three groups, along with variations in their proportions over time. These findings offer valuable insight into the relative proportions of different types of diagnostic delay. Such knowledge may help identify and address potential inefficiencies within the breast cancer screening process and the clinical diagnostic work-up.

**Implications of all the available evidence**

The majority of delays was due to misdiagnosis of screen-detected cancers at a previous screen, whereas interval cancers showed the worst survival. The proportions of women with misdiagnosis after recall and with missed interval cancers significantly declined over the years. Implementation of AI and other imaging modalities besides mammography at screening, on-going training and education of healthcare providers dedicated to breast care, and well-structured case discussions at multidisciplinary meetings may help to minimise the risk of misdiagnosis at screening and clinical breast imaging, expediting breast cancer confirmation in women participating at screening mammography programmes.

## Introduction

Breast cancer remains a leading cause of death in women, despite the implementation of screening mammography programmes and significant improvements in breast cancer therapy.^1^ Screening aims to detect breast cancer at an early stage, but some women participating at screening mammography may face a significant delay in their breast cancer diagnosis. This may be due to several causes, including cancers overlooked at screening mammography or a delay in breast cancer confirmation among recalled women with suspicious findings at screening mammography.^2^, ^3^, ^4^ In the latter situation, the principal causes identified were incorrect BI-RADS classification during clinical work-up and misinterpretation of breast imaging abnormalities by the radiologist. A smaller proportion of delays arose from biopsy-related errors, and in very rare cases delays occurred in patients who chose to discontinue their scheduled follow-up.^2^^,^^5^^,^^6^ Knowledge regarding survival outcomes in women with delayed breast cancer diagnosis remains limited. A Norwegian study indicated no significant difference between missed cancers and those diagnosed without delay, offering a moderately optimistic view regarding survival after a delayed breast cancer diagnosis.^7^

To our knowledge, data on the proportions of the various types of diagnostic breast cancer delay in a screened population is lacking, as well as information on the survival of these women. Therefore, we assessed to which degree women participating in the Dutch population-based screening programme experienced a delay in their breast cancer diagnosis after recall or were diagnosed with screen-detected breast cancers (SDC) or interval breast cancers (IC) that had been missed, respectively at their previous biennial screening session or at their latest screen. We determined the tumour characteristics, surgical therapy and survival rates of these three groups, as well as variations in their proportions over time.

## Methods

### Study population and screening procedure

We included all women who underwent screening in the South of The Netherlands between January 1, 1999 and January 1, 2019, with free biennial screening mammography offered from the age of 50–75 years. Prior to participation, women are asked for permission to use their data for quality assurance of the screening programme and scientific purposes. Three recalled women did not consent and were therefore excluded. Ethical approval was waived by the Dutch Central Committee on Research involving Human Subjects (CCMO).

Details of the screening programme have been published previously.^8^ In summary, all mammograms were obtained by specialised mammography radiographers at four screening units (one fixed unit and three mobile units). In 2009–2010, screen-film mammography was replaced by digital screening mammography. The mammograms were routinely double read by a team of certified screening radiologists, each of them reading more than 5000 screen yearly. Recalled women were assessed at specialised breast units at one of 15 regional or university hospitals.

### Follow-up and review of screen-detected breast cancers and interval breast cancers

During a period of at least two years (until the next biennial screening session), one of the screening radiologists and several radiology residents collected data on breast radiology, surgery and pathology at the hospitals where the assessment of recalled women took place.

For recalled women, a delay in breast cancer diagnosis was defined as the pathological confirmation of breast cancer more than three months after the initial assessment of the suspicious screening mammography abnormality at the breast unit. In case a women with SDC had experienced a false positive recall in the previous screening round (i.e., no breast cancer was diagnosed at work-up), two screening radiologists reviewed the screening mammograms to determine whether both recalls concerned the same lesion at screening mammography, implying an erroneous assessment of the first recall and thus resulting in a diagnostic delay of at least two years. The radiologists were blinded for each other's opinion and discrepancies were solved by consensus.

The screening mammograms of all women with breast cancer detected at subsequent screening rounds were also independently reviewed by two screening radiologists in order to determine whether the cancer was visible at the previous screen and should have been recalled at that time. The radiologist was initially blinded to the screening mammogram for which the women had been recalled and assessed the previous screening mammogram for suspicious abnormalities, using prior screening mammograms for comparison. In case of a suspicious lesion, the reviewer then determined whether it matched the recalled lesion. Each cancer was considered either to be missed, to show a minimal sign or to be occult/not present at the previous screen. Minimal sign lesions have a very low probability of malignancy of 0.5% and are therefore not recalled when detected at screening mammography.^9^ Consequently, we scored cancers with minimal sign lesions at the previous screen as ‘not missed’. In some cases, the radiologist only became aware of a lesion on the previous screening mammogram after subsequent comparison with the final screening mammogram that had resulted in a recall. In these cases, the final assessment was done through consensus reading with the other reviewer, irrespective of whether or not the lesion had been missed previously. Again, the radiologists were blinded for each other's opinion.

ICs are breast cancers diagnosed after a negative screen (i.e., no recall) and before the next scheduled screen. True ICs were not detectable at the previous screening round, unlike missed ICs, that were missed at the previous screening round. The invitation letter of the screenings programme advises women with breast symptoms to consult their general practitioner and not to participate in the screening. Other details on the methods of detecting ICs have been published previously.^10^ An interval of 30 months was used as the cut-off for the time between the last screening and the diagnosis of an interval cancer, thereby excluding women who missed a screening round from the interval cancer group. Opportunistic screening of asymptomatic women outside the framework of our nation-wide breast cancer screening programme occurs, but these women hardly ever also undergo biennial screening within the nation-wide screening programme. Cancers with a diagnostic delay in women detected through opportunistic screening are per definition not considered to be interval cancers within the context of the nation-wide screening programme. Since 2011, ICs have been identified by linkage of screening records to the Netherlands Cancer Registry (NCR), the Netherlands Comprehensive Cancer Organisation (IKNL), and regional pathology laboratories. Prior to 2011, this linkage was still under development, and data were supplemented through other tracing methods: 1) radiotherapy reports were obtained from regional institutes for women who received treatment for breast malignancy and participated in the screening programme, 2) pathology specimens from several regional pathology laboratories were requested a few months after a hospital had requested the screening mammograms of a woman not recalled for further analysis, 3) pathology records were collected if a woman cancelled a subsequent screening after being diagnosed with breast cancer following a previous negative screen, and 4) the screening centre periodically received reports on ICs from general practitioners or medical specialists. Only women who emigrated abroad were not captured through linkage with the Netherlands Cancer Registry(NCR), the Netherlands Comprehensive Cancer Organisation (IKNL) and the Regional Register of Death (Gemeentelijke Basisadministratie Persoonsgegevens).

All ICs were reviewed by the screening radiologists and classified as missed, showing a minimal sign or not being visible (either mammographically occult or not present) at the latest screening mammogram. The review process was similar to the one used for reviewing the cancers detected at subsequent screening, except that the screening mammogram was now compared with the diagnostic mammogram.

A total of six screening radiologists participated in the various review processes of the screening mammograms.

SDCs were divided in ductal carcinoma in-situ (DCIS) and invasive cancers. Lobular carcinoma in-situ (LCIS) was considered to be a benign lesion. The tumour size was determined from the pathology resection specimen. In the case of neo-adjuvant therapy, the tumour size was assessed at pre-operative breast imaging (usually breast MRI). Tumour grading was performed according to the modified Bloom & Richardson (B&R) grading system. Axillary lymph nodes were considered negative if they showed only isolated tumour cells or sub micro-metastasis (<0.2 mm).

Regular linkage to the Regional Register of Death (Gemeentelijke Basisadministratie Persoonsgegevens) was performed to determine the survival of women with SDCs or IC. The last linkage was conducted in January 2024. All patients had a minimum follow-up time of five years, except for the patients who died within this period.

### Statistical analysis

The Chi-square or Fisher's exact test was used when testing differences between the subgroups with respect to tumour characteristics and treatment. The Kaplan–Meier estimate was used to determine survival after delay in breast cancer diagnosis. In case of bilateral disease, the tumour with the most advanced tumour stage was included in the analysis. In case of multiple foci of cancer, only the largest tumour was taken into account. In the footnotes of the tables, we have specified whether or not missing values were included in the comparative analyses. No imputation methods were applied. For all analyses, a p-value less than 0.05 was considered statistically significant. Statistical analyses were performed using Statistical Package for Social Science 28.0 (IBM Corp. Released 2021. IBM SPSS Statistics for Windows, Version 28.0. Armonk, NY: IBM Corp).

### Role of the funding source

This research did not receive any funding.

## Results

### Overall screening outcome

**Fig. 1:**
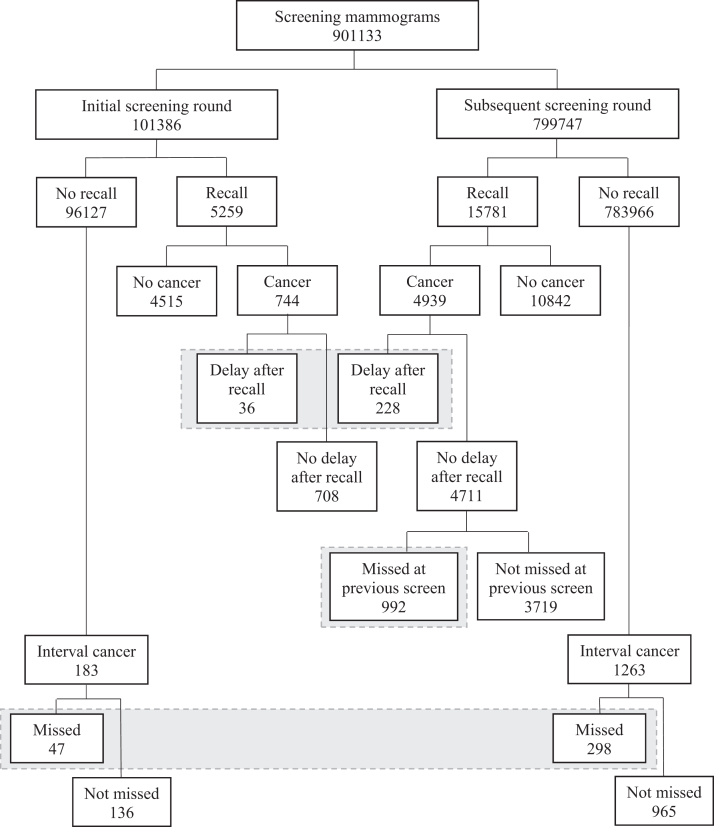
**Study population of women screened between January 1, 1999 and January 1, 2019**.

A total of 901,133 screening examinations were obtained between January 1999 and January 2019, including 101,386 initial screens and 799,747 subsequent screens (Fig. 1). The recall rate for initial screens was 5.2% (5259/101,386), with a cancer detection rate (CDR) of 7.3 per 1000 screens (744/101,386). Breast cancer was confirmed by biopsy more than three months after recall in 36 of the 744 (4.8%) women with SDC.

For subsequent screens, the recall rate was 2.0% (15,781/799,747) with a CDR of 6.2 per 1000 screens (4939/799,747). A total of 228 recalled women (4.6%, 228/4939)) experienced a delay in their breast cancer confirmation. In 21.1% (992/4711) of women without a diagnostic delay of their SDC after recall, the cancer was considered to be missed at the previous screen.

Among the initial screens, a total of 183 ICs were diagnosed (CDR of 1.8 per 1000 screens), of which 47 (25.7%) were considered to be missed at the latest screening examination. Following a subsequent screen, 1263 women were diagnosed with IC (1.6 per 1000 screens), of which 298 (23.6%) were visible and should have been referred at the latest screen.

Of the 6157 women of whom screening mammograms were reviewed (all interval cancers, and SDCs in a subsequent screening round without a delay in diagnostic work-up), six cases were identified in which the reviewing radiologists, after reviewing the previous screening mammogram and comparing it with the clinical mammogram, revised their original classification from ‘not missed/occult’ or ‘minimal sign’ to ‘missed’. This reclassification occurred after consensus was reached that the tumour was retrospectively visible on the previous mammogram.

In total, 0.2% of all recalled women were lost to follow-up (45/21,040) and no loss to follow-up was observed among women in the delay groups. The remaining recalled women underwent further diagnostic work-up. Of the 21,040 recalled women, 5683 (27.0%) were diagnosed with breast cancer. As noted, 264 experienced a delay in diagnostic work-up after recall, with 119 (45.1%) receiving only additional imaging. Among those without delay, 992 cancers had been missed in a previous round, of whom 974 (98.2%) underwent both additional imaging and biopsy. Of the 4427 women with breast cancer and no delay of any kind, 4308 (97.3%) received both imaging and biopsy (see Supplementary Figure S1). Altogether, 1601 women experienced a form of delay in their breast cancer diagnosis, comprising 83 women recalled after their initial screening (36 with a delay in diagnostic work-up after recall and 47 with a delay due to a missed IC) and 1518 women recalled after subsequent screening (228 with a delay in diagnostic work-up after recall, 992 with a delay due to a tumour missed at the previous screening, and 298 with a delay due to a missed IC) (Fig. 1). Consequently, 26.6% (1601/6028) of the women diagnosed with breast cancer in our study population, either SDC or emerging as IC, experienced a delay in their cancer diagnosis.

### Proportions of screen-detected cancers with diagnostic delay and interval cancers

The largest of the three groups with a delayed breast cancer diagnosis concerned women with SDCs missed at the previous screening round, comprising 14.5% (screening period 2004–2008) to 17.0% (2014–2018) of all SDCs and missed ICs (Table 1). This subgroup represented 62.0% (992/1601) of all diagnostic cancer delays, and corresponded to a rate of 0.11 per 1000 screening examinations. Missed ICs was the second largest group, with proportions significantly decreasing from 9.4% for women screened in 1999–2003 to 4.2% for women screened in 2014–2018 (p < 0.0001). These cases represented 21.5% (345/1601) of all diagnostic delays and corresponded to an incidence rate of 0.038 per 1000 screening examinations. Finally, the proportion of women who experienced a delayed cancer diagnosis after recall also significantly declined over the years, from 6.3% in 1999–2003 to 3.3% in 2014–2018 (p = 0.0028). This subgroup comprised 16.5% (264/1601) of all diagnostic delays and was associated with an incidence rate of 0.029 per 1000 screening examinations.

**Table 1:** Trends in the proportion and mortality rate per 1000 screens of screen-detected cancers with or without a diagnostic delay, screen-detected cancers missed at previous screening and missed interval cancers.

	Screening period 1999–2003	Screening period 2004–2008	Screening period 2009–2013	Screening period 2014–2018	Total	p-value^a^
**Screen-detected cancers without any diagnostic delay**^b^						
Number (%)	525 (67.9)	688 (73.3)	1462 (73.2)	1752 (75.5)	4427	0.0007
Mortality rate per 1000 screens	0.506	0.374	0.308	0.294		NA
**Delayed cancer diagnosis after recall**^c^						
Number (%)	49 (6.3)	45 (4.8)	93 (4.7)	77 (3.3)	264	0.0028
Mortality rate per 1000 screens	0.00147	0.00362	0.00186	0.00061		NA
**Screen-detected cancers missed at previous screening**^d^						
Number (%)	126 (16.3)	136 (14.5)	336 (16.8)	394 (17.0)	992	0.3157
Mortality rate per 1000 screens	0.00659	0.00603	0.00966	0.00485		NA
**Missed interval cancers**						
Number (%)	73 (9.4)	69 (7.4)	105 (5.3)	98 (4.2)	345	<0.0001
Mortality rate per 1000 screens	0.00879	0.00724	0.00409	0.00273		NA
Total	773	938	1996	2321	6028	

NA = not available/not applicable.

a The presented p-value reflects whether there are statistically significant differences in the proportions within the delay group across different time periods.

b This group contains all screen-detected cancers of women recalled after initial and subsequent screens of whom the cancer was detected without any delay.

c Pathological confirmation of breast cancer more than 3 months after recall.

d This group contains all screen-detected cancers of women recalled after subsequent screens of whom the cancer was missed at a previous screening.

### Tumour characteristics, surgical treatment and survival of women with a diagnostic breast cancer delay

**Fig. 2:**
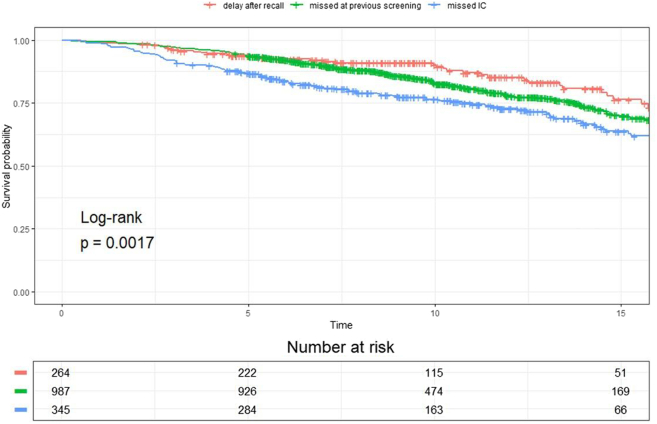
**Kaplan–Meier curves of the overall survival by year of women with a delay in diagnosis**. IC = Interval Carcinoma. Delay after recall: This group contains all screen-detected cancers of women recalled after initial and subsequent screens of whom the cancer was detected with a delay of at least three months. Missed at previous screening: This group contains all screen-detected cancers of women recalled after subsequent screens of whom the cancer was missed at a previous screening. Missed IC: This groups contains all women with interval cancers of whom the cancer was missed at a previous screening.

Missed ICs had the worst prognostic tumour characteristics compared to women with a diagnostic delay in diagnosis after recall and those with a SDC missed at the previous screening round, including a larger proportion of invasive cancers sized >20 mm (58.6% vs 19.1% and 23.2%, p < 0.0001), a higher risk of axillary lymph node metastases (49.7% vs 17.2% and 25.0%, p < 0.0001) and a higher proportion of B&R grade II or III disease (67.3% vs 54.0% and 50.2%, p < 0.0001, Table 2). Also, the missed ICs were more frequently ER-negative (p < 0.0001), PR-negative (p = 0.0018), Her2-negative (p = 0.021) and receptor triple-negative (p = 0.0062). Women with missed ICs more frequently underwent mastectomy (42.6%, 147/345) than women with SDCs that had been missed at the previous screen (19.3%, 191/992) or women with a delay in their breast cancer diagnosis after recall (20.1%, 53/264, p < 0.0001). The women with missed ICs had a significantly poorer overall survival than the women who experienced a delayed cancer diagnosis after recall and women with SDCs missed at the previous screening round (p = 0.0041 and p = 0.0107, respectively) (Fig. 2). In contrast, the women with missed ICs showed no significant difference in 5-year overall survival compared to true ICs (87.2% vs 87.8%, p = 0.78, Supplementary Table S2). Additional analyses comparing women with SDCs with or without a diagnostic delay after recall (divided by first and subsequent screens), as well as women with ICs either missed or not missed at the latest screen, are provided in Supplementary Tables S1 and S2 At first screening, no significant differences were found between women with or without a delay with respect to tumour characteristics and surgical treatment. However, in the group of subsequent screens, women without a delay more frequently presented with invasive carcinoma NST (78.4% vs 71.3%, p < 0.001) and were more likely to have axillary lymph node metastases (21.8% vs 15.4%, p = 0.042). Compared to women whose SDC had not been missed at a previous screen, those with SDCs missed at their previous screens more frequently presented with invasive cancer rather than ductal carcinoma in-situ (86.2 vs 80.1%, p < 0.001), had larger tumour sizes (T2+ stage 23.2% vs 18.2%, p = 0.001), higher probability of lymph node metastases (25.0% vs 20.8%, p = 0.008) and were more likely to undergo mastectomy (17.4% vs 14.0%, p = 0.023). Compared to women with ICs that had not been missed at the latest screening mammogram, those with missed ICs had significantly larger tumours (T2+ stage: 58.6% vs 50.2%, p = 0.008), more frequent showed lymph node metastases (49.7% vs 42.6%, p = 0.045), and were more likely to undergo mastectomy (42.6% vs 30.2%, p < 0.001).

**Table 2:** Tumour characteristics and survival of screen-detected cancers and interval cancers in a screened population.

	Screen-detected cancers without any diagnostic delay N = 4427	Delayed cancer diagnosis after recall^e^ N = 264	Screen-detected cancers missed at previous screening N = 992	Missed interval cancers N = 345	p-value^a^
Mean age, years	61.7	62.4	64.1	62.2	<0.001
Diagnostic delay, months	–	24.7	–f	12.4	NA
Type of cancer, n (%)					<0.0001
Ductal carcinoma in-situ	928 (21.0)	49 (18.6)	137 (13.8)	9 (2.6)	
Invasive cancer	3499 (79.0)	215 (81.4)	855 (86.2)	336 (97.4)	
Histological type of invasive cancers, n (%)					<0.0001^c^
Non-specific type (NST)	2764 (79.0)	154 (71.6)	654 (76.5)	243 (72.3)	<0.0001^b^
Lobular	421 (12.0)	34 (15.8)	111 (13.0)	59 (17.6)	
Mixed NST/lobular	120 (3.4)	1 (0.5)	35 (4.1)	20 (6.0)	
Other	189 (5.4)	24 (11.2)	55 (6.4)	12 (3.6)	
Unknown	5 (0.1)	2 (0.9)	–	2 (0.6)	
T-stage of invasive cancers, n (%)					<0.0001^c^
T1a-c (≤20 mm)	2807 (80.2)	173 (80.5)	655 (76.6)	136 (40.5)	<0.0001^b^
T2+ (>20 mm)	677 (19.3)	41 (19.1)	198 (23.2)	197 (58.6)	
Unknown	15 (0.4)	1 (0.5)	2 (0.2)	3 (0.9)	
Lymph node status of invasive cancers, n (%)					<0.0001
N+	788 (22.5)	37 (17.2)	214 (25.0)	167 (49.7)	
No	2597 (74.2)	170 (79.1)	613 (71.7)	164 (48.8)	
Unknown	114 (3.3)	8 (3.7)	28 (3.3)	5 (1.5)	
Modified B&R grading of invasive cancers, n (%)					<0.0001
I	1457 (41.6)	92 (42.8)	400 (46.8)	97 (28.9)	
II	1463 (41.8)	86 (40.0)	370 (43.3)	167 (49.7)	
III	452 (12.9)	30 (14.0)	59 (6.9)	59 (17.6)	
Unknown	127 (3.6)	7 (3.3)	26 (3.0)	13 (3.9)	
Estrogen receptor status of invasive cancers, n (%)					0.0001^c^
Positive	3086 (88.2)	195 (90.7)	792 (92.6)	283 (84.2)	<0.0001^b^
Negative	379 (10.8)	18 (8.4)	58 (6.8)	52 (15.5)	
Unknown	34 (1.0)	2 (0.9)	5 (0.6)	1 (0.3)	
Progesteron receptor status of invasive cancers, n (%)					0.0087^c^
Positive	2512 (71.8)	155 (72.1)	632 (73.9)	215 (64.0)	0.0018^b^
Negative	945 (27.0)	58 (27.0)	217 (25.4)	120 (35.7)	
Unknown	42 (1.2)	2 (0.9)	6 (0.7)	1 (0.3)	
Her2/Neu receptor status of invasive cancers, n (%)					0.021
Positive	273 (7.8)	15 (7.0)	58 (6.8)	39 (11.6)	
Negative	2666 (76.2)	160 (74.4)	655 (76.6)	229 (68.2)	
Unknown	560 (16.0)	40 (18.6)	142 (16.6)	68 (20.2)	
Triple negative receptor status of invasive cancers, n (%)					0.0062
Yes	222 (6.3)	11 (5.1)	30 (3.5)	28 (8.3)	
No	3188 (91.1)	201 (93.5)	809 (94.6)	298 (88.7)	
Unknown	89 (2.5)	3 (1.4)	16 (1.9)	10 (3.0)	
Final surgical treatment, n (%)					<0.0001
Breast conserving surgery	3610 (81.5)	204 (77.3)	791 (79.7)	190 (55.1)	
Mastectomy	763 (17.2)	53 (20.1)	191 (19.3)	147 (42.6)	
No surgery/unknown	54 (1.2)	7 (2.7)	10 (1.0)	8 (2.3)	
5-year survival, % (95% CI)	93.9 (93.2–94.6)	93.8 (0.9–96.8)	93.8 (92.3–95.3)	86.9 (83.4–90.5)	0.0017^d^

B&R = Bloom & Richardson.

a p-values calculated when comparing columns 3–5 only (i.e., excluding screen-detected cancers without delay).

b Patient with unknown values were excluded from the analyses.

c Fisher's exact test.

d Logrank-test.

e Pathological confirmation of breast cancer more than 3 months after recall.

f As this pertains to a tumour that was missed during a previous screening round, the diagnostic delay is estimated to be approximately 24 months. Nevertheless, precise data regarding the duration of the delay are lacking.

### Trends in tumour characteristics, surgical treatment and survival of women with a diagnostic breast cancer delay

For women with a delay in their breast cancer diagnosis after recall, we found no significant trends over time or proportional fluctuations in tumour characteristics, surgical treatment or survival, except for a positive progesterone receptor status decreasing from 87.2% (1999–2003) to 64.6% (2009–2013, Supplementary Table S3). For women with SDCs missed at the previous biennial screening, the proportion of DCIS increased from 6.3% (8/126) in 1999–2003 to 16.0% (63/394) in 2014–2018 (p = 0.041, Supplementary Table S4). Among the invasive cancers, the lobular type increased from 8.5% (10/118) in 1999–2003 to 17.5% (58/331) in 2014–2018 (p = 0.0041) and we found a shift in B&R tumour grading over time, with grade I/II cases increasing from 83.0% (83/100) in 1999–2003 to 94.5% in 2014–2018 (312/330, p = 0.0057). For missed ICs, all parameters remained comparable through the years (Supplementary Table S5).

## Discussion

We found that 26.6% of women who underwent biennial screening mammography experienced a delay in their breast cancer diagnosis, of which SDCs missed at the previous screening round comprised the largest group (62.0%). In every aspect, missed ICs showed the poorest prognostic features and they were characterised by the highest mastectomy rate and poorest survival prognosis. Changes over time in tumour characteristics were mainly observed in recalled women with breast cancer missed at the previous biennial screen.

A delay in breast cancer diagnosis at screening mammography may be due to several factors and intervention on these can potentially improve screening outcome. The delay may be the result of extremely dense breast tissue masking the breast malignancy at mammography or caused by detection errors or interpretation errors by the screening radiologist.^3^^,^^4^ Before we performed the current study, we conducted an investigation within the same study population to identify the specific causes of diagnostic delay among the subgroup of women who experienced a delay in diagnostic work-up after recall.^2^ The primary causes were found to be an incorrect BI-RADS classification during the clinical diagnostic work-up, as well as misinterpretation of abnormalities by the clinical radiologist. A smaller proportion of delays were attributable to diagnostic errors in biopsy procedures. Only a very small number of patients discontinued follow-up or further diagnostic assessment on their own initiative.^2^^,^^5^^,^^6^ Implementation of breast tomosynthesis, contrast-enhanced mammography or contrast-enhanced breast MRI in a screening mammography programme has been proven to enhance the cancer detection rate, especially in women with extremely dense breast tissue, and to reduce the number of ICs.^11^, ^12^, ^13^ Nevertheless, these diagnostic tools have not yet been implemented in the Dutch screening programme; however, their effectiveness in this population is currently under investigation.^14^^,^^15^ Furthermore, recent studies have shown that artificial intelligence (AI) supported mammography screening, which is similarly not yet implemented in the Dutch breast cancer screening programme, may result in comparable screening outcomes while lowering the workload.^16^^,^^17^ Given the rapidly expanding body of evidence and the increasing number of publications on this topic, its implementation is anticipated in the foreseeable future. The selective use of these imaging modalities and AI, in addition to or replacing standard mammography screening, are promising developments to reduce the number of ICs and breast cancers that have been missed, at respectively the latest or previous screening round. However, AI and these novel imaging modalities also pose a risk of overdiagnosis (defined as the identification of a condition that would not have caused clinical symptoms or harm if left undetected), and potentially overtreatment of indolent lesions.^18^

Our observation that 20%–25% of the SDCs and ICs were missed at screening and should have been recalled at an earlier stage is in line with other studies.^7^^,^^19^ In a subanalysis, we demonstrated that there was no significant difference in 5-year overall survival between missed and true ICs. This finding aligns with a Norwegian study, which also reported no significant difference in overall survival between true, minimal signs, and missed ICs (p = 0.43), as well as SDCs (p = 0.82).^7^ Nevertheless, missed ICs were associated with larger tumour size, a higher incidence of axillary lymph node metastases, and a greater likelihood of undergoing mastectomy compared to true ICs, reflecting a greater disease burden. Furthermore, missed ICs exhibited the poorest prognostic tumour characteristics, the highest mastectomy rate, and significantly worse overall survival in our study when compared to both missed SDCs and cancers in women who experienced a diagnostic delay following recall. False-negative results in breast cancer screening may place women at risk by delaying care for symptomatic lesions, as a consequence of false reassurance. The screening provider (in the Dutch context, the government) bears an ethical responsibility to mitigate such risks, ensuring the programme does not inflict unnecessary harm. Encouragingly, the prognosis of women with IC has improved dramatically since 1995.^20^ Our current finding that the improved increase in 5-year survival from 83.6% for women screened in 1999–2003 to 90.8% for women screened in 2014–2018 was not statistically significant may be explained by the fact that we only included missed ICs for survival analysis, in combination with a follow-up time restricted to 5-year survival of a relatively small group of patients.

A timely confirmation of breast cancer after recall is of utmost importance in order to maximise the efficacy of a screening programme. Although the proportion of women who faced a diagnostic delay after recall significantly declined through the years, one out of 30 recalled women is still experiencing such a delay. Causes leading to initial misdiagnosis are multifactorial and may especially be related to the radiologists, using the Breast Imaging Reporting and Data System (BI-RADS) score incorrectly, the type of tumour, false negative biopsy results and lack of communication with other specialists.^2^^,^^4^ In the Netherlands, screening radiologists attend quality sessions every three months, supervised by a coordinating screening radiologist, in which they discuss the screening mammography features of ICs and SDCs and confronting the radiologists with cancers missed at screening. Moreover, since the start of 2024 screening radiologists get feedback on their individual screening performance at regular intervals, which is benched to that of their fellow screeners. This feedback may help radiologists to adjust, for example, their recall rate if this falls beyond a predefined range and probably gives them tools to increase their screening performance.

Changes over time with respect to tumour characteristics were mainly found in SDCs missed at the previous screen. The significant increase in the proportion of DCIS, from 6.3% to 16.0%, is probably due to the overall increase of DCIS detected in the screened population, especially after the transition from screen-film screening to digital screening.^21^ We have no clear explanation for the increase of invasive lobular cancers, although this increase becomes less profound when the mixed NST/lobular cancers are added to the pure lobular cancers. Also, the histopathological diagnosis of invasive lobular cancer may have changed over time.^22^ The shift to more low grade invasive cancers may be partially explained by the simultaneous increase of invasive lobular cancers, which are generally better differentiated but larger at the time of diagnosis than invasive cancers of the non-specific type.^23^

The length of a delay in breast cancer confirmation has an impact on morbidity as we found that women with a delayed diagnosis of SDCs at subsequent screening rounds more frequently underwent mastectomy than women without a delayed diagnosis. Moreover, women with missed SDCs at prior screenings had a higher proportion of invasive tumours, larger tumour sizes, an increased chance of lymph node involvement, and higher mastectomy rates compared to women with SDCs that had not been missed. These results are consistent with other studies of screened populations demonstrating a relationship between the presence of a diagnostic delay (type of delay not specified) and increased tumour size, as well as an higher risk of lymph nodes and the presence of distant metastases.^24^^,^^25^ Consequently, the treatment of women with breast cancer missed at their previous screen is more aggressive compared to those without a delay in diagnosis. These findings underscore the clinical relevance of an effective diagnostic process and a correct assessment of the abnormalities seen on mammography, as delays may contribute to a more advanced disease and thus an increased treatment burden.

A limitation of our study is the review process used to evaluate whether breast cancers had been missed at screening mammography. Ideally, a case mix of mammograms with and without breast cancers would have been preferable as the reviewing radiologists would then not have been biased by the fact that all women were ultimately diagnosed with breast cancer. However, taking into account the resulting number of cases to be reviewed, this kind of review would not have been be feasible in daily practice. A form of retrospective knowledge bias was introduced in six of 6157 cases during the review process, as the reviewing radiologists revised their initial classification–from ‘not missed/occult’ or ‘minimal sign’ to ‘missed’–after re-assessing the prior screening mammogram in light of the clinical mammogram. This reclassification occurred after consensus was reached that the tumour was retrospectively visible on the previous mammogram. Given the small number of such cases, any potential impact on the overall study results is considered negligible. Another limitation of our study is the absence of data regarding disease-specific survival. However, the primary focus of this investigation is not on survival outcomes, but rather on tumour characteristics (including stage) and surgical treatment.

In summary, we found that a majority of delays was due to misdiagnosis of SDCs at a previous screen, whereas missed ICs showed the worst survival. Nevertheless, missed ICs and non-missed ICs demonstrated similar 5-year overall survival. The proportions of women with misdiagnosis after recall and with missed ICs significantly declined over the years. These findings suggest that while delays in breast cancer diagnosis remain detrimental, their clinical consequences are becoming increasingly manageable. Implementation of AI and other imaging modalities besides mammography at screening, on-going training and education of healthcare providers dedicated to breast care, and well-structured case discussions at multidisciplinary meetings may help to minimise the risk of misdiagnosis at screening and clinical breast imaging, expediting breast cancer confirmation in women participating at screening mammography programmes.

## Contributors

Lucien EM Duijm: Conceptualisation, Data curation, Formal analysis, Investigation, Methodology, Project administration, Resources, Supervision, Validation, Visualisation, Writing-original draft.

Eline L van der Veer: Conceptualisation, Validation, Writing-review and editing.

Hermen C van Beek: Writing-review and editing.

Wikke Setz-Pels: Writing-review and editing.

Vivian van Breest Smallenburg: Writing-review and editing.

Rob MG van Bommel: Writing-review and editing.

Clemence L op de Coul-Froger: Writing-review and editing.

Maaike Gielens: Writing-review and editing.

Dominique JP van Uden: Writing-review and editing.

Adri C Voogd: Conceptualisation, Formal analysis, Supervision, Validation, Writing-review and editing.

## Data sharing statement

The data that support the findings of this study are available from the corresponding author upon reasonable request.

## Declaration of interests

The authors of this manuscript have no disclosures.
