# Supplementary material for: Frequency and survival of delayed breast cancer diagnosis in women participating at screening mammography in the Netherlands: a population-based study
Source: Lancet Reg Health Eur. 2025 Nov 14;61:101526. doi: 10.1016/j.lanepe.2025.101526 (PMC12662070; doi:10.1016/j.lanepe.2025.101526)
Supplement: Supplementary Figure and Tables [file mmc1.docx]

**Supplementary Figure 1. Initial assessment of recalled women, screened between January 1, 1999 and January 1, 2019.**


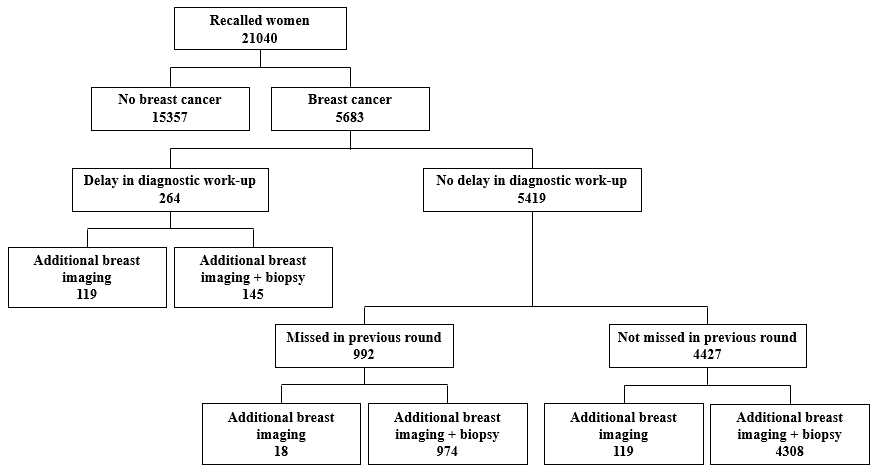


**Supplementary Table 1. Tumour characteristics and survival of the screen-detected cancers (after recall-first screen, after recall-subsequent screen and at previous**

**screen) with versus without delay.**

|  | **Breast cancer diagnosis after recall, first screen^±^** | | | **Breast cancer diagnosis after recall, subsequent screen^±^** | | | **Screen-detected cancer at previous screen** | | |
| --- | --- | --- | --- | --- | --- | --- | --- | --- | --- |
|  | **Delay**  **N = 36** | **No delay**  **N = 708** | **P-value** | **Delay**  **N = 228** | **No delay**  **N = 4711** | **P-value** | **Missed**  **N = 992** | **Not missed**  **N = 3719** | **P-value** |
| Type of cancer, n (%)  Ductal carcinoma in-situ  Invasive cancer | 8 (22·2)  28 (77·8) | 189 (26·7)  519 (73·3) | 0·55 | 40 (17·5)  188 (82·5) | 876 (18·6)  3835 (81·4) | 0·69 | 137 (13·8)  855 (86·2) | 739 (19·9)  2980 (80·1) | <0·001 |
| Histological type of invasive cancers, n (%)  Non-specific type (NST)  Lobular  Mixed NST/lobular  Other  Unknown | 19 (67·9)  5 (17·9)  -  3 (10·7)  1 (3·6) | 410 (79·0)  62 (11·9)  23 (4·4)  24 (4·6)  - | 0·21 | 134 (71·3)  29 (15·4)  2 (1·1)  22 (11·7)  1 (0·5) | 3008 (78·4)  470 (12·3)  133 (3·5)  220 (5·7)  4 (0·1) | <0·001 | 654 (76·5)  111 (13·0)  35 (4·1)  55 (6·4)  - | 2354 (79·1)  359 (12·0)  98 (3·3)  165 (5·5)  4 (0·1) | 0·37 |
| T-stage of invasive cancers, n (%)  T1a-c (<20 mm)  T2+ (>20 mm)  Unknown | 18 (64·3)  8 (28·6)  2 (7·1) | 378 (72·8)  136 (26·2)  5 (1·0) | 0·63 | 155 (82·4)  32 (17·0)  1 (0·5) | 3084 (80·5)  739 (19·3)  12 (0·3) | 0·45 | 655 (76·6)  198 (23·2)  2 (0·2) | 2429 (81·5)  541 (18·2)  10 (0·3) | 0·001 |
| Lymph node status of invasive cancers, n (%)  N+  No  Unknown | 8 (28·6)  18 (64·3)  3 (7·1) | 168 (32·4)  331 (63·8)  20 (3·9) | 0·76 | 29 (15·4)  152 (80·9)  7 (3·7) | 834 (21·7)  2879 (75·1)  122 (3·2) | 0·042 | 214 (25·0)  613 (71·7)  28 (3·3) | 620 (20·8)  2266 (76·0)  94 (3·2) | 0·008 |
| Modified B&R grading of invasive cancers, n (%)  I  II  III  Unknown | 10 (35·7)  10 (35·7)  5 (17·9)  3 (10·7) | 236 (45·5)  199 (38·3)  53 (10·2)  31 (6·0) | 0·35 | 83 (44·1)  76 (40·4)  25 (13·3)  4 (2·1) | 1621 (42·3)  1634 (42·6)  459 (12·0)  121 (3·1) | 0·75 | 400 (46·8)  370 (43·3)  59 (6·9)  26 (3·0) | 1221 (41·0)  1264 (42·4)  400 (13·4)  95 (3·2) | <0·001 |
| Estrogen receptor status of invasive cancers, n (%)  Positive  Negative  Unknown | 24 (85·7)  2 (7·1)  2 (7·1) | 482 (92·9)  26 (5·0)  11 (2·1) | 0·64^#^ | 172 (91·5)  16 (8·5)  - | 3396 (88·6)  410 (10·7)  29 (0·7) | 0·33 | 792 (92·6)  58 (6·8)  5 (0·6) | 2604 (87·4)  352 (11·8)  24 (0·8) | <0·001 |
| Progesteron receptor status of invasive cancers, n (%)  Positive  Negative  Unknown | 19 (67·9)  7 (25·0)  2 (7·1) | 428 (82·5)  79 (15·2)  12 (2·3) | 0·17^#^ | 137 (72·9)  51 (27·1)  - | 2716 (70·8)  1081 (28·2)  38 (1·0) | 0·69 | 632 (73·9)  217 (25·4)  6 (0·7) | 2084 (69·9)  864 (29·0)  32 (1·1) | 0·034 |
| Her2/Neu receptor status of invasive cancers, n (%)  Positive  Negative  Unknown | 2 (7·1)  19 (67·9)  7 (25·0) | 39 (7·5)  377 (72·6)  103 (19·8) | 0·98^#^ | 13 (6·9)  141 (75·0)  34 (18·1) | 292 (7·6)  2943 (76·7)  600 (15·7) | 0·80 | 58 (6·8)  655 (76·6)  142 (16·6) | 234 (7·9)  2288 (76·8)  458 (15·3) | 0·35 |
| Triple negative receptor status of invasive cancers, n (%)  Yes  No  Unknown | 2 (7·1)  24 (85·7)  2 (7·1) | 10 (1·9)  495 (95·4)  14 (2·7) | 0·11^#^ | 9 (4·8)  178 (94·7)  1 (0·5) | 242 (6·3)  3502 (91·3)  91 (2·4) | 0·37 | 30 (3·5)  809 (94·6)  16 (1·9) | 212 (7·1)  2693 (90·4)  75 (2·5) | <0·001 |
| Final surgical treatment, n (%)  Breast conserving surgery  Mastectomy  No surgery/unknown | 28 (77·8)  8 (22·2)  - | 508 (71·8)  190 (26·8)  10 (1·4) | 0·62^#^ | 183 (80·3)  38 (16·7)  7 (3·1) | 3964 (84·1)  693 (14·7)  54 (1·1) | 0·024 | 809 (81·6)  173 (17·4)  10 (1·0) | 3155 (84·8)  520 (14·0)  44 (1·2) | 0·023 |

B&R = Bloom & Richardson

^±^Pathological confirmation of breast cancer more than 3 months after recall.

^#^Fisher’s exact test

**Supplementary Table 2.** **Tumour characteristics, treatment and survival of interval carcinomas with versus without missed lesions on previous screens**

|  | **Interval carcinoma**  **missed**  **N = 345** | **Interval carcinoma**  **not missed**  **N = 1101** | **P-value** |
| --- | --- | --- | --- |
| Type of cancer, n (%)  Ductal carcinoma in-situ  Invasive cancer | 9 (2·6)  336 (97·4) | 53 (4·8)  1048 (95·2) | 0·078 |
| Histological type of invasive cancers, n (%)  Non-specific type (NST)  Lobular  Mixed NST/lobular  Other  Unknown | 243 (72·3)  59 (17·6)  20 (6·0)  12 (3·6)  2 (0·6) | 779 (74·3)  175 (16·7)  36 (3·4)  53 (5·1)  5 (0·5) | 0·14 |
| T-stage of invasive cancers, n (%)  T1a-c (<20 mm)  T2+ (>20 mm)  Unknown | 136 (40·5)  197 (58·6)  3 (0·9) | 510 (48·7)  526 (50·2)  12 (1·1) | 0·008 |
| Lymph node status of invasive cancers, n (%)  N+  No  Unknown | 167 (49·7)  164 (48·8)  5 (1·5) | 446 (42·6)  565 (53·9)  37 (3·5) | 0·045 |
| Modified B&R grading of invasive cancers, n (%)  I  II  III  Unknown | 97 (28·9)  167 (49·7)  59 (17·6)  13 (3·9) | 266 (25·4)  449 (42·8)  285 (27·2)  48 (4·6) | 0·001 |
| Estrogen receptor status of invasive cancers, n (%)  Positive  Negative  Unknown | 283 (84·2)  52 (15·5)  1 (0·3) | 792 (75·7)  254 (24·2)  2 (0·3) | <0·001 |
| Progesteron receptor status of invasive cancers, n (%)  Positive  Negative  Unknown | 215 (64·0)  120 (35·7)  1 (0·3) | 604 (57·6)  442 (42·2)  2 (0·2) | 0·037 |
| Her2/Neu receptor status of invasive cancers, n (%)  Positive  Negative  Unknown | 39 (11·6)  229 (68·2)  68 (20·2) | 123 (11·7)  748 (71·4)  177 (16·9) | 0·86 |
| Triple negative receptor status of invasive cancers, n (%)  Yes  No  Unknown | 28 (8·3)  298 (88·7)  10 (3·0) | 146 (13·9)  862 (82·3)  40 (3·8) | 0·006 |
| Final surgical treatment, n (%)  Breast conserving surgery  Mastectomy  No surgery/unknown | 190 (55·1)  147 (42·6)  8 (2·3) | 742 (67·4)  333 (30·2)  26 (2·4) | <0·001 |
| 5-year overall survival, n (%) | 301 (87·2) | 967 (87·8) | 0·78 |

B&R = Bloom & Richardson

**Supplementary Table 3. Trends in tumour characteristics and treatment of women with delayed breast cancer diagnosis after recall^±^**

|  | **Screening period**  **1999-2003**  **N = 49** | **Screening period**  **2004-2008**  **N = 45** | **Screening period**  **2009-2013**  **N = 93** | **Screening period**  **2014-2018**  **N = 77** | **P-value*** |
| --- | --- | --- | --- | --- | --- |
| Type of cancer, n (%)  Ductal carcinoma in-situ  Invasive cancer | 10 (20·4)  39 (79·6) | 10 (22·2)  35 (77·8) | 14 (15·1)  79 (84·9) | 15 (19·5)  62 (80·5) | 0·727 |
| Histological type of invasive cancers, n (%)  Non-specific type (NST)  Lobular  Mixed NST/lobular  Other  Unknown | 30 (76·9)  5 (12·8)  -  4 (10·3)  - | 26 (74·3)  4 (11·4)  1 (2·9)  2 (5·7)  2 (5·7) | 59 (74·7)  11 (13·9)  -  9 (11·4)  - | 39 (62·9)  14 (22·6)  -  9 (14·5)  - | 0·859^#^ |
| T-stage of invasive cancers, n (%)  T1a-c (<20 mm)  T2+ (>20 mm)  Unknown | 32 (82·1)  7 (17·9)  - | 28 (80·0)  6 (17·1)  1 (2·9) | 59 (74·7)  20 (25·3)  - | 54 (87·1)  8 (12·9)  - | 0·310 |
| Lymph node status of invasive cancers, n (%)  N+  No  Unknown | 7 (17·9)  30 (76·9)  2 (5·1) | 9 (25·7)  24 (68·6)  2 (5·7) | 16 (20·3)  63 (79·7)  - | 5 (8·1)  53 (85·5)  4 (6·5) | 0·127 |
| Modified B&R grading of invasive cancers, n (%)  I  II  III  Unknown | 17 (43·6)  16 (41·0)  1 (2·6)  5 (12·8) | 15 (42·9)  13 (37·1)  5 (14·3)  2 (5·7) | 34 (43·0)  30 (38·0)  15 (19·0)  - | 26 (41·9)  27 (43·5)  9 (14·5)  - | 0·516 |
| Estrogen receptor status of invasive cancers, n (%)  Positive  Negative  Unknown | 38 (97·4)  -  1 (2·6) | 27 (77·1)  7 (20·0)  1 (2·9) | 72 (91·1)  7 (8·9)  - | 58 (93·5)  4 (6·5)  - | 0·055^#^ |
| Progesteron receptor status of invasive cancers, n (%)  Positive  Negative  Unknown | 34 (87·2)  4 (10·3)  1 (2·6) | 24 (68·6)  10 (28·6)  1 (2·9) | 51 (64·6)  28 (35·4)  - | 46 (74·2)  16 (25·8)  - | 0·042 |
| Her2/Neu receptor status of invasive cancers, n (%)^$^  Positive  Negative  Unknown | -  6 (15·4)  33 (84·6) | 4 (11·4)  26 (74·3)  5 (14·3) | 7 (8·9)  71 (89·9)  1 (1·3) | 4 (6·5)  57 (91·9)  1 (1·6) | 0·565 |
| Triple negative receptor status of invasive cancers, n (%)^$^  Yes  No  Unknown | -  38 (97·4)  1 (2·6) | 2 (5·7)  31 (88·6)  2 (5·7) | 5 (6·3)  74 (93·7)  - | 4 (6·5)  58 (93·5)  - | 0·910# |
| Final surgical treatment, n (%)  Breast conserving surgery  Mastectomy  No surgery/unknown | 40 (81·6)  9 (18·4)  - | 31 (68·9)  13 (28·9)  1 (2·2) | 72 (77·4)  19 (20·4)  2 (2·1) | 61 (79·2)  12 (15·6)  4 (5·2) | 0·380 |

B&R = Bloom & Richardson

^±^Pathological confirmation of breast cancer more than 3 months after recall

*Patient with unknown values were excluded from the analyses

^#^Fisher’s Exact test

^$^Screening period 1999-2003 was not included in the analyses

**Supplementary Table 4. Trends in tumour characteristics and treatment of women with screen-detected cancers missed at previous screening**

|  | **Screening period**  **1999-2003**  **N = 126** | **Screening period**  **2004-2008**  **N = 136** | **Screening period**  **2009-2013**  **N = 336** | **Screening period**  **2014-2018**  **N = 394** | P-value* |
| --- | --- | --- | --- | --- | --- |
| Type of cancer, n (%)  Ductal carcinoma in-situ  Invasive cancer | 8 (6·3)  118 (93·7) | 16 (11·8)  120 (88·2) | 50 (14·9)  286 (85·1) | 63 (16·0)  331 (84·0) | 0·041 |
| Histological type of invasive cancers, n (%)  Non-specific type (NST)  Lobular  Mixed NST/lobular  Other  Unknown | 94 (79·7)  10 (8·5)  8 (6·8)  6 (5·1)  - | 89 (74·2)  16 (13·3)  5 (4·2)  10 (8·3)  - | 225 (78·7)  27 (9·4)  18 (6·3)  16 (5·6)  - | 246 (74·3)  58 (17·5)  4 (1·2)  23 (6·9)  - | 0·0041 |
| T-stage of invasive cancers, n (%)  T1a-c (<20 mm)  T2+ (>20 mm)  Unknown | 89 (75·4)  29 (24·6)  - | 86 (71·7)  33 (27·5)  1 (0·8) | 221 (77·3)  64 (22·4)  1 (0·3) | 259 (78·2)  72 (21·8)  - | 0·578 |
| Lymph node status of invasive cancers, n (%)  N+  No  Unknown | 34 (28·8)  84 (71·2)  - | 31 (25·8)  86 (71·7)  3 (2·5) | 72 (25·2)  208 (72·7)  6 (2·1) | 77 (23·3)  235 (71·0)  19 (5·7) | 0·852 |
| Modified B&R grading of invasive cancers, n (%)  I  II  III  Unknown | 46 (39·0)  37 (31·4)  17 (14·4)  18 (15·3) | 59 (49·2)  50 (41·7)  6 (5·0)  5 (4·2) | 132 (46·2)  134 (46·9)  18 (6·3)  2 (0·7) | 163 (49·2)  149 (45·0)  18 (5·4)  1 (0·3) | 0·0057 |
| Estrogen receptor status of invasive cancers, n (%)  Positive  Negative  Unknown | 108 (91·5)  9 (7·6)  1 (0·9) | 112 (93·3)  6 (5·0)  2 (1·7) | 261 (91·3)  24 (8·4)  1 (0·3) | 311 (94·0)  19 (5·7)  1 (0·3) | 0·487 |
| Progesteron receptor status of invasive cancers, n (%)  Positive  Negative  Unknown | 88 (74·6)  29 (24·6)  1 (0·9) | 89 (74·2)  28 (23·3)  3 (2·5) | 209 (73·1)  76 (26·6)  1 (0·3) | 246 (74·3)  84 (25·4)  1 (0·3) | 0·943 |
| Her2/Neu receptor status of invasive cancers, n (%)^$^  Positive  Negative  Unknown | -  1 (0·9)  117 (99·2) | 7 (5·8)  90 (75·0)  23 (19·2) | 23 (8·0)  262 (91·6)  1 (0·3) | 28 (8·5)  302 (91·2)  1 (0·3) | 0·921 |
| Triple negative receptor status of invasive cancers, n (%)^$^  Yes  No  Unknown | -  108 (91·5)  10 (8·5) | 2 (1·7)  114 (95·0)  4 (3·3) | 15 (5·2)  270 (94·4)  1 (0·3) | 13 (3·9)  317 (95·8)  1 (0·3) | 0·264 |
| Final surgical treatment, n (%)  Breast conserving surgery  Mastectomy  No surgery/unknown | 101 (80·2)  25 (19·8)  - | 105 (77·2)  29 (21·3)  2 (1·5) | 265 (78·9)  66 (19·6)  5 (1·5) | 320 (81·2)  71 (18·0)  3 (0·8) | 0·828 |

B&R = Bloom & Richardson, NA = not available/not applicable.

*Patients with unknown values were excluded from the analyses

^$^Screening period 1999-2003 was not included in the analyses

**Supplementary Table 5. Trends in tumour characteristics and treatment of women with missed interval breast cancers**

|  | **Screening period**  **1999-2003**  **N = 73** | **Screening period**  **2004-2008**  **N = 69** | **Screening period**  **2009-2013**  **N = 105** | **Screening period**  **2014-2018**  **N = 98** | **P-value*** |
| --- | --- | --- | --- | --- | --- |
| Type of cancer, n (%)  Ductal carcinoma in-situ  Invasive cancer | 1 (1·4)  72 (98·6) | 2 (2·9)  67 (97·1) | 4 (3·8)  101 (96·2) | 2 (2·0)  96 (98·0) | 0·949^#^ |
| Histological type of invasive cancers, n (%)  Non-specific type (NST)  Lobular  Mixed NST/lobular  Other  Unknown | 51 (70·8)  16 (22·2)  2 (2·8)  2 (2·8)  1 (1·4) | 46 (68·7)  11 (16·4)  8 (11·9)  1 (1·5)  1 (1·5) | 72 (71·3)  15 (14·9)  9 (8·9)  5 (5·0)  - | 74 (77·1)  17 (17·7)  1 (1·0)  4 (4·2)  - | 0·363^#^ |
| T-stage of invasive cancers, n (%)  T1a-c (<20 mm)  T2+ (>20 mm)  Unknown | 26 (36·1)  45 (62·5)  1 (1·4) | 33 (49·3)  33 (49·3)  1 (1·5) | 39 (38·6)  61 (60·4)  1 (1·0) | 38 (39·6)  58 (60·4)  - | 0·389 |
| Lymph node status of invasive cancers, n (%)  N+  No  Unknown | 36 (50·0)  35 (48·6)  1 (1·4) | 31 (46·3)  35 (52·2)  1 (1·5) | 53 (52·5)  47 (46·5)  1 (1·0) | 47 (49·0)  47 (49·0)  2 (2·1) | 0·899 |
| Modified B&R grading of invasive cancers, n (%)  I  II  III  Unknown | 19 (26·4)  27 (37·5)  15 (20·8)  11 (15·3) | 27 (40·3)  34 (50·7)  5 (7·5)  1 (1·5) | 27 (26·7)  55 (54·6)  18 (17·8)  1 (1·0) | 24 (25·0)  51 (53·1)  21 (21·9)  - | 0·095 |
| Estrogen receptor status of invasive cancers, n (%)  Positive  Negative  Unknown | 60 (83·3)  11 (15·3)  1 (1·4) | 57 (85·1)  10 (14·9)  - | 88 (87·1)  13 (12·9)  - | 78 (81·3)  18 (18·8)  - | 0·724 |
| Progesteron receptor status of invasive cancers, n (%)  Positive  Negative  Unknown | 49 (68·1)  22 (30·6)  1 (1·4) | 44 (65·7)  23 (34·2)  - | 69 (68·3)  32 (31·7)  - | 53 (55·2)  43 (44·8)  - | 0·179 |
| Her2/Neu receptor status of invasive cancers, n (%)^$^  Positive  Negative  Unknown | 2 (2·8)  4 (5·6)  66 (91·7) | 12 (17·9)  53 (79·1)  2 (3·0) | 9 (8·9)  92 (91·1)  - | 16 (16·7)  80 (83·3)  - | 0·151 |
| Triple negative receptor status of invasive cancers, n (%)^$^  Yes  No  Unknown | -  62 (86·1)  10 (13·9) | 5 (7·5)  62 (92·5)  - | 11 (10·9)  90 (89·1)  - | 12 (12·5)  84 (87·5)  - | 0·586 |
| Final surgical treatment, n (%)  Breast conserving surgery  Mastectomy  No surgery/unknown | 43 (58·9)  28 (38·4)  2 (2·7) | 44 (63·8)  24 (34·8)  1 (1·4) | 53 (50·5)  50 (47·6)  2 (1·9) | 50 (51·0)  45 (45·9)  3 (3·1) | 0·264 |

B&R = Bloom & Richardson

*Patient with unknown values were excluded from the analyses

^#^Fisher’s Exact test

^$^Screening period 1999-2003 was not included in the analyses
